# Molecular Characterization of SehB, a Type II Antitoxin of *Salmonella enterica* Serotype Typhimurium: Amino Acid Residues Involved in DNA-Binding, Homodimerization, Toxin Interaction, and Virulence

**DOI:** 10.3389/fmicb.2020.00614

**Published:** 2020-04-09

**Authors:** Fernando Chimal-Cázares, Gabriela Hernández-Martínez, Sabino Pacheco, Miguel A. Ares, Jorge Soria-Bustos, Manuel Sánchez-Gutiérrez, Jeannett A. Izquierdo-Vega, Jose Antonio Ibarra, Jorge A. González-y-Merchand, Jean-Pierre Gorvel, Stéphane Méresse, Miguel A. De la Cruz

**Affiliations:** ^1^Unidad de Investigación Médica en Enfermedades Infecciosas y Parasitarias, Hospital de Pediatría, Centro Médico Nacional Siglo XXI, Instituto Mexicano del Seguro Social, Mexico City, Mexico; ^2^Departamento de Microbiología, Escuela Nacional de Ciencias Biológicas, Instituto Politécnico Nacional, Mexico City, Mexico; ^3^Departamento de Microbiología Molecular, Instituto de Biotecnología, Universidad Nacional Autónoma de México, Cuernavaca, Mexico; ^4^Instituto de Ciencias de la Salud, Universidad Autónoma del Estado de Hidalgo, Pachuca, Mexico; ^5^Aix Marseille Univ, CNRS, INSERM, CIML, Marseille, France

**Keywords:** SehB, SehAB, toxin-antitoxin, *Salmonella*, virulence

## Abstract

*Salmonella enterica* serotype Typhimurium is a bacterium that causes gastroenteritis and diarrhea in humans. The genome of *S.* Typhimurium codes for diverse virulence factors, among which are the toxin-antitoxin (TA) systems. SehAB is a type II TA, where SehA is the toxin and SehB is the antitoxin. It was previously reported that the absence of the SehB antitoxin affects the growth of *S.* Typhimurium. In addition, the SehB antitoxin can interact directly with the SehA toxin neutralizing its toxic effect as well as repressing its own expression. We identified conserved residues on SehB homologous proteins. Point mutations were introduced at both N- and C-terminal of SehB antitoxin to analyze the effect of these changes on its transcription repressor function, on its ability to form homodimers and on the virulence of *S.* Typhimurium. All changes in amino acid residues at both the N- and C-terminal affected the repressor function of SehB antitoxin and they were required for DNA-binding activity. Mutations in the amino acid residues at the N-terminal showed a lower capacity for homodimer formation of the SehB protein. However, none of the SehB point mutants were affected in the interaction with the SehA toxin. In terms of virulence, the eight single-amino acid mutations were attenuated for virulence in the mouse model. In agreement with our results, the eight amino acid residues of SehB antitoxin were required for its repressor activity, affecting both homodimerization and DNA-binding activity, supporting the notion that both activities of SehB antitoxin are required to confer virulence to *Salmonella enterica*.

## Introduction

*Salmonella enterica* comprises Gram-negative bacteria with around 2500 serotypes responsible for severe gastroenteritis and systemic infections in warm-blooded animals, including humans (Haraga et al., 2008; Jajere, 2019). By horizontal gene transfer, *S. enterica* has evolved to adapt to both extracellular and intracellular conditions, expressing a myriad of virulence factors, such as secretion systems, adhesins, flagella, and toxins, among others (Fabrega and Vila, 2013). In this way, toxin-antitoxin systems have emerged as important elements that affect the pathogenicity of many bacteria, regulating their physiology, persistence and virulence (Lobato-Marquez et al., 2016; Harms et al., 2018; Ronneau and Helaine, 2019). Six types of TA systems are currently described, classified mainly according to the ribonucleic or proteinaceous structure of the antitoxin and how it antagonizes the cognate toxin (Page and Peti, 2016). In type I and III, the antitoxin is an RNA molecule, which directly binds to RNA and protein, respectively, while in the others (II, IV, V, and VI), the antitoxin is a protein that directly or indirectly neutralizes the expression or activity of the toxin (Lobato-Marquez et al., 2016). Especially in the type II TA systems, both toxin and antitoxin are proteins and are transcriptionally organized in a bicistronic operon, where mostly the antitoxin gene is upstream of the toxin gene (Yamaguchi and Inouye, 2009).

While type II toxins possess a variety of enzymatic functions (ribonuclease, kinase, adenylyl-, and acetyl-transferase) (Mutschler et al., 2011; Yamaguchi et al., 2011; Harms et al., 2015; Cheverton et al., 2016; Rocker and Meinhart, 2016), type II antitoxins interact with the cognate toxins and also act as transcription repressors of their own expression (Yamaguchi and Inouye, 2011). Most type II antitoxins possess a DNA-binding domain located at the N-terminal and a region involved in homodimerization at the C-terminal (Chan et al., 2016). The *higBA* (host inhibition of growth) TA genes code for type II systems, which are widely prevalent in bacteria (Pandey and Gerdes, 2005), where HigB is a toxin that cleaves mRNAs in the presence of ribosomes, and HigA is the antitoxin that neutralizes the toxic effect of HigB (Hurley and Woychik, 2009). Contrary to other type II TA genes, in the *higBA* bicistronic operon the *higB* toxin gene is upstream of the *higA* antitoxin gene. Moreover, HigA-type antitoxins in *Escherichia coli* and *Shigella flexneri*, unlike *Proteus vulgaris* and *Pseudomonas aeruginosa*, present an unusual organization possessing the homo-heterodimerization region and DNA-binding domain at the N- and C-terminal, respectively (Schureck et al., 2014; Yang et al., 2016; Liu et al., 2019; Xu et al., 2019; Yoon et al., 2019).

In *Salmonella enterica* serotype Typhimurium (*S*. Typhimurium), SehAB is a type II TA system homologous to HigBA, where SehA is the toxin and SehB is the antitoxin. SehB protein contains 142 amino acids and has a molecular weight and isoelectric point of 15.92 kDa and 5.56, respectively. SehB antitoxin represses its own expression by direct binding to its promoter region, and a SehB homodimer was observed in solution (De la Cruz et al., 2013). In terms of virulence, the absence of SehB dramatically affects the virulence of *S*. Typhimurium in mice (De la Cruz et al., 2013).

In this work we identified conserved residues of the SehB antitoxin by alignment of amino acid sequence of homologous proteins. Eight plasmid constructs expressing point mutations of the SehB protein located at the N- and C-terminal were generated. All of these mutant proteins were unable to bind to their promoter region, thus affecting the ability of the SehB antitoxin to repress its own expression. This DNA-binding activity was impaired both due to the lack of SehB homodimerization caused by mutations at the N-terminal and by alterations of the helix-turn-helix (HTH)-type domain located at the C-terminal. All SehB amino acid tested were dispensable for the interaction with the SehA toxin. Interestingly, these amino acid residues of the SehB antitoxin were required for the virulence of *S*. Typhimurium. The functional characterization of the SehB protein showed that Y32, L42, L52, I60, S107, L121, L129, and F140 amino acids are involved in different molecular mechanisms of this protein and they are important for *S*. Typhimurium virulence.

## Materials and Methods

### Bacterial Strains and Growth Conditions

Bacterial strains used in this study are listed in Table 1. Strains were cultured in LB broth. Ampicillin (200 μg/ml), kanamycin (50 μg/ml), tetracycline (10 μg/ml), and chloramphenicol (34 μg/ml) were added when required. Bacterial suspensions were prepared from overnight LB broth cultures. Then, 250 ml-flasks containing 50 ml of LB broth were inoculated with bacterial suspensions that presented an initial OD_600 nm_ of 0.05. These cultures were incubated at 37°C in a shaking incubator at 200 rpm.

**TABLE 1:** Bacterial strains and plasmids used in this study.

Strain or plasmid	Genotype or description	References or source
***S*. typhimurium strains**		
12023	Wild-type, virulent	De la Cruz et al., 2013
Δ*sehB*	12023 Δ*sehB*:FRT	De la Cruz et al., 2013
***E. coli***		
MC4100	Cloning strain	Casadaban, 1976
SU101	Reporter strain of the LexA based genetic system for homodimerization assays; Km^*R*^	Dmitrova et al., 1998
SU202	Reporter strain of the LexA based genetic system for heterodimerization assays; Km^*R*^	Dmitrova et al., 1998
BL21 (DE3)	Strain for expression of recombinant proteins	Invitrogen
**Plasmids**		
*sehAB-gfp*	pFPV25 derivative containing the *sehAB* promoter region	De la Cruz et al., 2013
pMPM-K6	p15A derivative cloning vector, pBAD (*ara*) promoter; Km^*R*^	Mayer, 1995
pK6-SehB-WT	pMPM-K6 derivative expressing His_6_-SehB-WT from the pBAD (*ara*) promoter	This study
pK6-SehB-Y32A	pMPM-K6 derivative expressing His_6_-SehB-Y32A from the pBAD (*ara*) promoter	This study
pK6-SehB-L42A	pMPM-K6 derivative expressing His_6_-SehB-L42A from the pBAD (*ara*) promoter	This study
pK6-SehB-L52A	pMPM-K6 derivative expressing His_6_-SehB-L52A from the pBAD (*ara*) promoter	This study
pK6-SehB-I60A	pMPM-K6 derivative expressing His_6_-SehB-I60A from the pBAD (*ara*) promoter	This study
pK6-SehB-S107A	pMPM-K6 derivative expressing His_6_-SehB-S107A from the pBAD (*ara*) promoter	This study
pK6-SehB-L121A	pMPM-K6 derivative expressing His_6_-SehB-L121A from the pBAD (*ara*) promoter	This study
pK6-SehB-L129A	pMPM-K6 derivative expressing His_6_-SehB-L129A from the pBAD (*ara*) promoter	This study
pK6-SehB-F140A	pMPM-K6 derivative expressing His_6_-SehB-F140A from the pBAD (*ara*) promoter	This study
pSR658	Vector expressing LexA_DBDwt_ for homodimerization assays; Tc^*R*^	Daines and Silver, 2000
pSR658-H-NS	pSR658 derivative expressing LexA_DBDwt_-H-NS; Tc^*R*^	Martinez et al., 2011
pSR658-SehB-WT	pSR658 derivative expressing LexA_DBDwt_-SehB-WT; Tc^*R*^	This study
pSR658-SehB-Y32A	pSR658 derivative expressing LexA_DBDwt_-SehB-Y32A; Tc^*R*^	This study
pSR658-SehB-L42A	pSR658 derivative expressing LexA_DBDwt_-SehB-L32A; Tc^*R*^	This study
pSR658-SehB-L52A	pSR658 derivative expressing LexA_DBDwt_-SehB-L52A; Tc^*R*^	This study
pSR658-SehB-I60A	pSR658 derivative expressing LexA_DBDwt_-SehB-I60A; Tc^*R*^	This study
pSR658-SehB-S107A	pSR658 derivative expressing LexA_DBDwt_-SehB-S107A; Tc^*R*^	This study
pSR658-SehB-L121A	pSR658 derivative expressing LexA_DBDwt_-SehB-L121A; Tc^*R*^	This study
pSR658-SehB-L129A	pSR658 derivative expressing LexA_DBDwt_-SehB-L129A; Tc^*R*^	This study
pSR658-SehB-F140A	pSR658 derivative expressing LexA_DBDwt_-SehB-F140A; Tc^*R*^	This study
pSR658-HilD	pSR658 derivative expressing LexA_DBDwt_-HilD; Tc^*R*^	Paredes-Amaya et al., 2018
pSR659	Vector expressing LexA_DBDmut_ for heterodimerization assays; Ap^*R*^	Daines and Silver, 2000
pSR659-HilE	pSR659 derivative expressing the LexA_DBDmut_-HilE fusion; Ap^*R*^	Paredes-Amaya et al., 2018
pSR659-SehA	pSR659 derivative expressing the LexA_DBDmut_-SehA fusion; Ap^*R*^	This study

### Protein Structure Determination

Amino acid sequence of SehB (STM4030.S) and SehA (STM4031) of *S.* Typhimurium strain LT2 was used to find templates on SWISS-MODEL server (Waterhouse et al., 2018). The toxin-antitoxin HigBA from *S. flexneri* (PDB code: 5YCL), which had the highest score of Sequence Coverage, Identity, GMQE (Global Model Quality Estimation) and Quaternary Structure Quality Estimate (QSQE), was chosen to build the 3D-structure of SehB monomeric by homology modeling. The models of SehAB toxin-antitoxin complex and SehB homodimer were built by superimposing of structures on HigBA complex and HigA homodimer. Figures were prepared using PyMol software.

### Construction of Plasmids

Plasmids and primers used in this study are listed in Tables 1, 2, respectively. To construct the plasmid pK6-SehB-WT, which carries a His_6_-tag at N-terminal of SehB protein, a fragment of the *sehB* gene was amplified by PCR from the pK6-SehB plasmid, using the primers pairs His_6_-SehB-*Nco*I-5′ and SehB-*Hin*dIII-3′. Site-specific mutations in *sehB* gene were introduced by overlapping PCR as previously described (Ho et al., 1989). Briefly, pairs of complementary oligonucleotides were designed (Table 2), and both mutagenic primers were combined in parallel PCRs with primers His_6_-SehB-*Nco*I-5′ and SehB-*Hin*dIII-3′, respectively, using pK6-SehB-WT plasmid as a template. The resulting PCR products were purified and mixed for a second PCR round with primers His_6_-SehB-*Nco*I-5′ and SehB-*Hin*dIII-3′, which allow the amplification of the entire *sehB* gene. The final PCR products were purified and digested with *Nco*I and *Hin*dIII and cloned into pMPM-K6 previously digested with the same enzymes, rendering plasmids pK6-SehBY-32A, pK6-SehB-Y42A, pK6-SehB-L52A, pK6-SehB-I60A, pK6-SehB-S107A, pK6-SehB-L121A, pK6-SehB-L129A, and pK6-SehB-F140A. To generate plasmids pSR658-SehB-WT, pSR658-SehB-Y32A, pSR658-SehB-Y42A, pSR658-SehB-L52A, pSR658-SehB-I60A, pSR658-SehB-S107A, pSR658-SehB-L121A, pSR658-SehB-L129A, and pSR658-SehB-F140A, fragments of the *sehB* gene were amplified by PCR with the primers pair SehB-*Xho*I-F/SehB-*Kpn*I-R, using as template each construction that carries the *sehB* point mutation including the wild-type *sehB* gene. The PCR products were digested with *Xho*I and *Kpn*I and cloned into the vector pSR658 previously digested with the same restriction enzymes. To generate the pSR659-SehA plasmid, the *sehA* gene was amplified by PCR using the primers SehA-*Xho*I-F/SehA-*Kpn*I-R. The resulting PCR product was digested with *Xho*I and *Kpn*I and cloned into the vector pSR659 digested with the same restriction enzymes. All constructs were verified by DNA sequencing.

**TABLE 2:** Oligonucleotides used in this study.

Oligonucleotide	Sequence (5′–3′)^*a*^
	**For mutagenesis**
sehB-Y32A-5′	GGGGAAGACCGAAATGACAATGAGGCACGCAGGG CACTAGCGCTAGTGGAG
sehB-Y32A-3′	CTCCACTAGCGCTAGTGCCCTGCGTGCCTCATTGT CATTTCGGTCTTCCCC
sehB-L42A-5′	AGGGCACTAGCGCTAGTGGAGTTTGCAGTCGACCA CGACGATCTTGAAAAC
sehB-L42A-3′	GTTTTCAAGATCGTCGTGGTCGACTGCAAACTCCA CTAGCGCTAGTGCCCT
sehB-L52A-5′	GACCACGACGATCTTGAAAACCCAGCATTTGAATTGC TCTGTGCCCGAATC
sehB-L52A-3′	GATTCGGGCACAGAGCAATTCAAATGCTGGGTTTTC AAGATCGTCGTGGTC
sehB-I60A-5′	CTATTTGAATTGCTCTGTGCCCGAGCAAGTGAATAC GAAAAACATGCGCCG
sehB-I60A-3′	CGGCGCATGTTTTTCGTATTCACTTGCTCGGGCACAGA GCAATTCAAATAG
sehB-S107A-5′	GCAGATCTTGCCAACGAACTTGGTGCAAAATCGAAC GTCAGCAACATCTTA
sehB-S107A-3′	TAAGATGTTGCTGACGTTCGATTTTGCACCAAGTTC GTTGGCAAGATCTGC
sehB-L121A-5′	AACATCTTAAATGGCCGCAGAGCAGCAACGGTTAA TCATATTAAAGCGCTT
sehB-L121A-3′	AAGCGCTTTAATATGATTAACCGTTGCTGCTCTGC GGCCATTTAAGATGTT
sehB-L129A-5′	CTAACGGTTAATCATATTAAAGCGGCAACACAACGC TTCAAACTACCAGCA
sehB-L129A-3′	TGCTGGTAGTTTGAAGCGTTGTGTTGCCGCTTTAATA TGATTAACCGTTAG
sehB-F133A-5′	CATATTAAAGCGCTTACACAACGCGCAAAACTACCAG CAGATGCCTTCATC
sehB-F133A-3′	GATGAAGGCATCTGCTGGTAGTTTTGCGCGTTGTG TAAGCGCTTTAATATG
sehB-F140A-5′	CGCTTCAAACTACCAGCAGATGCCGCAATCGAGTAG TTGGATATGTCCAGC
sehB-F140A-3′	GCTGGACATATCCAACTACTCGATTGCGGCATCTGCTGG TAGTTTGAAGCG
	**For cloning^*b*^**
His_6_-SehB-*Nco*I-5′	GGGCCATGGATCATCATCATCATCATCATGCAA CCAGCGCAAAAAAG
SehB-*Hin*dIII-3′	TCGAAGCTTATGAAGAGGGCGGG
SehB-*Xho*I-5′	TAACTCGAGATGGGAACTCATCGTCAGATGGA
SehB-*Kpn*I-3′	CATGGTACCCTACTCGATGAAGGCATCTGCT
SehA-*Xho*I-5′	AACCTCGAGATGGAACTACATTGTGCATGT
SehA-*Kpn*I-3′	GATGGTACCTCATTCTTTATTACCCCGATAGTATGC
	**For QPCR**
sehA-5′	ACCGTTCAATGAAGCGATGC
sehA-3′	TCGGCATGGGAAACGATATG
rrsH-5′	AGGCCTTCGGGTTGTAAAGT
rrsH-3′	ATTCCGATTAACGCTTGCAC
	**For EMSA**
GFP-5′	CTAAGAAACCATTATTATCATGAC
GFP-3′	TTCAACAAGAATTGGGACAACTCC

*The sequence corresponding to the mutagenized codon^*a*^ or restriction site^*b*^ is underlined.*

### Flow Cytometry Analysis of Bacteria

Bacteria grown in LB broth were treated and analyzed by flow cytometry as previously described (Aussel et al., 2011).

### Quantitative RT-PCR

Total RNA was extracted from bacteria grown under different culture conditions using the hot phenol method (Jahn et al., 2008). DNA was removed with TURBO DNA-free (Ambion, Inc.) and the quality of RNA was assessed using a NanoDrop (ND-1000; Thermo Scientific) and an Agilent 2100 bioanalyzer with a Picochip (Agilent Technologies). The absence of contaminating DNA was controlled by the lack of amplification products after 35 qPCR cycles using RNA as template. Control reactions with no template (water) and with no reverse transcriptase were run in all experiments. cDNA synthesis and qPCR were performed as described (Matter et al., 2018; Ares et al., 2019). Specific primers were designed with the Primer3Plus software and they are listed in Table 2. 16S rRNA (*rrsH*) was used as a reference gene for normalization and the relative gene expression was calculated using the 2^–ΔΔ*Ct*^ method (Livak and Schmittgen, 2001).

### Protein Purification

His_6_-SehB proteins were purified as previously reported (De la Cruz et al., 2013). Briefly, *E. coli* BL21(DE3) carrying the pK6-SehB plasmids (Table 1) was grown to mid-logarithmic phase. L(+)-arabinose (Sigma-Aldrich) was added to a final concentration of 0.1%, and the bacteria were grown at 6 h at 30°C. Subsequently, cells were pelleted by centrifugation, resuspended in urea buffer (8 M urea, 100 mM NaH_2_PO_4_ and 10 mM Tris-HCl, pH 8.0) and disrupted by sonication. This suspension was centrifuged, and the supernatant was filtered through a Ni-nitrilotriacetic acid agarose column (Qiagen) pre-equilibrated with urea buffer. After extensive washing with binding buffer containing 50 mM imidazole (200 ml), proteins were eluted with 500 mM imidazole (10 ml). Fractions were analyzed by SDS-PAGE. Protein concentration was determined by the Bradford procedure. Aliquots of each purified protein were stored at −70°C.

### Electrophoretic Mobility Shift Assay (EMSA)

EMSA experiments were performed as described previously (De la Cruz et al., 2007). PCR product corresponding to *sehAB* promoter region was amplified from *sehAB-gfp* plasmid using primers GFP-5′ and GFP-3′, which hybridize to the pFPV25 vector. This fragment (100 ng), was mixed with increasing concentrations of wild-type and mutants His_6_-SehB proteins in PBS/50% glycerol. They were incubated 30 min at room temperature and then separated by electrophoresis in 6% polyacrylamide gels in Tris-borate-EDTA buffer. DNA bands were visualized by staining with ethidium bromide.

### Western Blotting

Equal numbers of bacteria were used to prepare whole cell extracts and subjected to SDS-PAGE in 12% polyacrylamide gels and electroblotted onto PVDF membranes. After blocking with 5% non-fat milk, membranes were incubated with anti-His_6_ (abcam) and anti-LexA (Merck) polyclonal antibodies at 1:3000 and 1:10,000 dilutions, respectively. Goat anti-rabbit IgG (Sigma-Aldrich) conjugated to horseradish peroxidase (1:10,000) was used as secondary antibody, and the reactions were visualized with chemiluminescence reagents (Perkin Elmer).

### SehB Dimerization Assays

To test homodimerization of SehB, the pSR658, pSR658-H-NS, pSR658-SehB-WT, pSR658-SehB-Y32A, pSR658-SehB-Y42A, pSR658-SehB-L52A, pSR658-SehB-I60A, pSR658-SehB-S107A, pSR658-SehB-L121A, pSR658-SehB-L129A, and pSR658-SehB-F140A plasmids, were transformed into the *E. coli* SU101 reporter strain for homodimerization assays, which carries the chromosomal *sulA–lacZ* transcriptional fusion (Dmitrova et al., 1998). Transformants were grown in LB with tetracycline and 1 mM IPTG to induce expression of LexA_DBDwt_-SehB fusion proteins. Samples were collected at an OD_600 nm_ of 1.0 and used for the determination of β-galactosidase activity.

### SehB-SehA Heterodimerization Assays

To test the heterodimerization between SehA and SehB, the *E. coli* SU202 reporter strain (used for heterodimerization tests), which carries the *sulA–lacZ* transcriptional fusion with a hybrid LexA operator (Dmitrova et al., 1998) was first transformed with the plasmid pSR659-SehA, and then transformed with the pSR658-SehB-WT and mutagenic variants. Transformants were grown in LB with tetracycline and ampicillin and 1 mM IPTG was used to induce expression of LexA_DBDwt_ and LexA_DBDmut_ fusion proteins. Samples were collected at an OD_600 nm_ of 1.0 and used for the determination of β-Galactosidase activity.

### β-Galactosidase Assays

The β-Galactosidase assay and protein quantification to calculate specific activities were performed as previously described (Oropeza et al., 1999).

### Intracellular Replication Assays

RAW264.7 (ATCC TIB-71) mouse macrophages were seeded at a density of 10^6^ cells per well in 24-well tissue culture plates 24 h before use. Bacteria were cultured overnight at 37°C with shaking and were opsonized in DMEM containing FBS and 10% normal mouse serum for 30 min on ice. When pK6-SehB plasmids were used, we did not induce *sehB* expression from the arabinose promoter since the leaky expression that occurs is enough to complement the absence of *sehB* in the chromosome. Bacteria were added to cells at a multiplicity of infection (MOI) of 100. Plates were centrifuged at 500 *g* for 5 min at 4°C and incubated for 30 min at 37°C under an humidified 5% CO_2_ atmosphere. Cells were washed thrice with DMEM containing 100 μg/ml gentamicin and then incubated in this medium for 1 h to eliminate extracellular bacteria; after, the gentamicin concentration was decreased to 10 μg/ml for the remainder of the experiment. For enumeration of intracellular bacteria, macrophages were washed thrice with PBS and lysed with 0.1% Triton X-100 for 15 min, and 10-fold serial dilutions were plated onto LB agar plates to determine total CFUs. Plates were incubated overnight at 37°C, and colonies were counted. Each time point was performed in triplicate, and each experiment was performed three times.

### Survival Curves of Mice Infected With *S*. Typhimurium

Six- to eight-week-old Balb/c female mice were perorally inoculated with 10^5^ CFUs of WT *S.* Typhimurium or Δ*sehB* mutant transformed with different plasmids expressing both wild-type and mutants SehB proteins. When pK6-SehB plasmids were used, we did not induce *sehB* expression from the arabinose promoter since the leaky expression that occurs is enough to complement the absence of *sehB* in the chromosome. *S.* Typhimurium was prepared by growing 1 ml of each culture in 100 ml of LB broth for 3 h (OD_600 nm_ = 1.0) at 37°C, followed by plating 10^3^–10^6^ serial dilutions in triplicate on LB agar, and placed in a 37°C incubator overnight. CFUs were enumerated the following day, and the dose was appropriately prepared to the desired concentration. Infected mice were monitored at least every 12 h during 14 days, and moribund animals were sacrificed by CO_2_ asphyxiation at the end of the experiment. Survival was recorded as percentage of survival every day after infection. Survival analysis was conducted using the Kaplan-Meier survival test.

### Competitive Index

Competitive index experiments were performed as previously described (De la Cruz et al., 2013). Six Balb/c mice (6–8 weeks old) were inoculated perorally with equal amounts of two bacterial strains for a total of 10^5^ bacteria per mouse. When pK6-SehB plasmids were used, we did not induce *sehB* expression from the arabinose promoter since the leaky expression that occurs is enough to complement the absence of *sehB* in the chromosome. The spleens were harvested 2 days after inoculation, and homogenized. Bacteria were recovered and enumerated after plating a dilution series onto LB agar with the appropriate antibiotics. Competitive indexes (CI) were determined for each mouse (Beuzon and Holden, 2001).

### Statistical Analysis

All data were the averages of three independent experiments performed by triplicate. Data were analyzed with the GraphPad Prism 5.0 software (GraphPad Inc., San Diego, CA, United States) using two-tailed Student’s *t*-test. *P-*values of < 0.05 were considered significant.

## Results

### Identification of Conserved Amino Acid Residues in SehB Homologous Proteins

**FIGURE 1:**
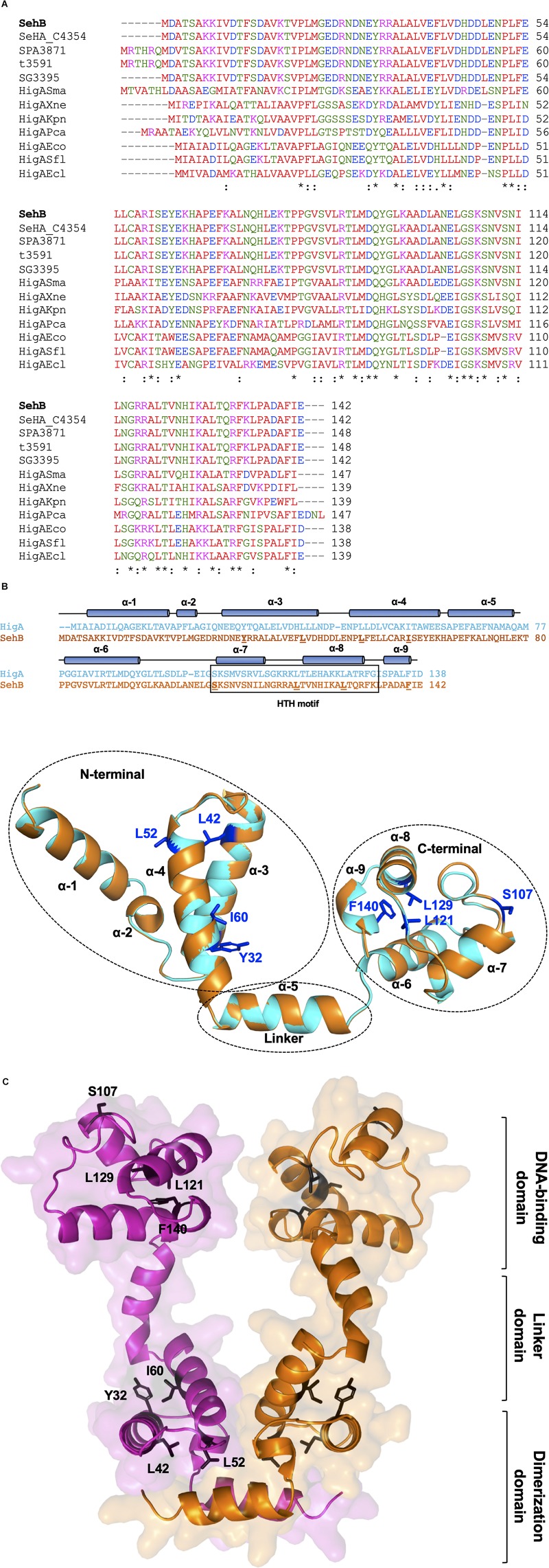
SehB homologs and structure prediction. **(A)** The amino acid sequence multialignment of SehB (STM4030.S) from *S.* Typhimurium strain LT2 (AAL22869.1) and homologs proteins SeHA_C4354 from *S.* Heidelberg strain SL476 (ACF69262.1), SPA3871 from *S.* Paratyphi A strain ATCC 915 (AAV79638.1), t3591 from *S.* Typhi strain Ty2 (AAO71094.1), SG3395 from *S.* Gallinarum strain 287/91 (CAR39186.1), HigASma from *S. marcescens* strain ATCC 13880 (KFD15043.1), HigAXne from *X. nematophila* strain ATCC 19061 (CBJ92634.1), HigAKpn from *K. pneumoniae* strain NTUH-K2044 (BAH66031.1), HigAPca from *P. carotovorum* strain ICMP 5702 (KML71864.1), HigAEco from *E. coli* strain MG1655 (NP_417553.1), HigASfl from *S. flexneri* strain 2457T (AAP18407.1) and HigAEcl from *E. cloacae* strain ATCC 13047 (YP_003613619.1), was performed using the Clustal Omega software (https://www.ebi.ac.uk/Tools/msa/clustalo/). Identical and similar residues are marked by asterisk and two points, respectively. Hydrophobic, polar, acidic and basic amino acids are colored in red, green, blue and magenta, respectively. **(B)** Schematic representation of the SehB predicted tertiary structure using the SWISS-MODEL server (Waterhouse et al., 2018). The cylinders represent alpha helices. Amino acids replaced by alanine are underlined. Putative HTH DNA-binding domain correspond to helices α-7 and α-8. Amino acids selected to be mutagenized are shown in the tertiary structure of SehB. **(C)** Model of SehB homodimer by superposition of monomeric structures on dimeric HigA using PyMol software. Both N- and C-terminal domains are depicted in the figure. Y32, L42, L52, I60, S107, L121, L129, and F140 residues are indicated in one SehB monomer. SehB monomers are colored in magenta and orange.

We began this study by searching homolog proteins of SehB antitoxin in different bacteria. Uncharacterized SehB homologs are present in serotypes of *Salmonella enterica* (Heidelberg, Paratyphi, Typhi, and Gallinarum), as well as in in other bacteria species such as *Serratia marcescens*, *Xenorhabdus nematophila*, *Klebsiella pneumoniae*, *Pectobacterium carotovora*, *E. coli*, *S. flexneri*, and *Enterobacter cloacae* (Figure 1A). By alignment of the amino acid sequence of this protein with other homologous SehB antitoxins, conserved amino acids were identified throughout the entire protein (Figure 1A). In addition, an *in silico* characterization of the SehB antitoxin identified both N- and C-terminal domains, which correspond to the 1-80 and 81-142 amino acid residues, respectively. We used as reference the HigA protein from *E. coli* K-12 and *S. flexneri*, which both show 46% identity and 67% similarity to SehB (Figure 1B). Each N- and C-terminal domain of SehB contain four α-helices, which correspond to a putative dimerization and a DNA binding domain, respectively, connected by the helix α5 (Figures 1B,C). Specifically, a helix-turn-helix (HTH) DNA-binding motif was found at the C-terminal from S107 to K134 amino acids (Figure 1C). Moreover, a 3D-model showed the SehB homodimer formation, displaying intermolecular interactions between both SehB monomers. To investigate further the functional role of conserved amino acids residues on the SehB antitoxin, alanine substitutions were performed at four residues of N- and C-terminal: Y32, L42, L52, I60, S107, L121, L129, and F140.

### Point Mutations at Both N- and C-Terminal Affect the Repressor Function of SehB

**FIGURE 2:**
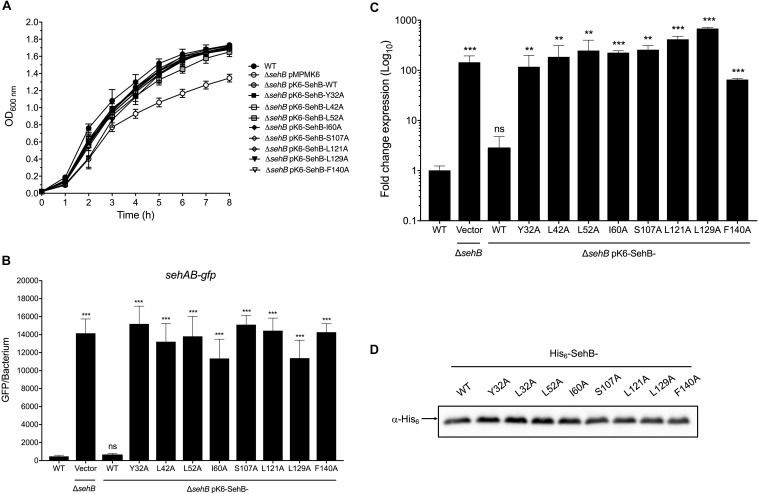
Effect of SehB point mutations on the transcription repression of *sehAB*. **(A)** Growth kinetics of *S*. Typhimurium wild-type (WT), Δ*sehB* pMPM-K6 and Δ*sehB* expressing SehB-WT and SehB point mutants. Bacteria were grown at 37°C in LB broth and the optical density was monitored at 600 nm. **(B)** *S*. Typhimurium wild-type (WT), Δ*sehB* pMPM-K6 and Δ*sehB* expressing the SehB-WT and SehB point mutants carrying a transcriptional fusion *sehAB-gfp* were grown in LB broth and samples were taken at a final OD_600__*nm*_ of 1.5. The relative fluorescence intensity of bacteria was determined by flow cytometry. 10,000 bacteria were analyzed to calculate the mean GFP/bacterium. **(C)** Transcription of *sehA* gene in LB broth at early stationary phase (OD_600 nm_ = 1.5) at 37°C was quantified by RT-qPCR. These graphics represent the mean of three separate experiments performed with triplicate samples with standard deviations. **(D)** His_6_-SehB-WT and His_6_-SehB-point mutations fusion proteins were analyzed by Western Blotting using a polyclonal anti-His_6_ antibody. Statistically significant with respect to WT *S*. Typhimurium; **p < 0.01; ***p < 0.001; ns, not significant.

We previously reported that SehB antitoxin acts as a transcriptional repressor of its own expression (De la Cruz et al., 2013). In order to investigate further the role of amino acids on SehB transcription activity, we generated eight plasmid constructions, each of them containing the *sehB* gene with single point mutations. Hence, these plasmids were introduced into the Δ*sehB* mutant and the repressor function of the SehB antitoxin on its own expression was evaluated using *sehAB-gfp* plasmid fusion. Of note, we did not induce *sehB* expression from the arabinose promoter since the leaky expression that occurs is enough to complement the absence of *sehB* in the chromosome (Figure 2). All of these strains displayed growth similar to that of the SehB-WT strain, indicating that mutations in the N- and C-terminal domains of SehB did not affect the function of the SehB protein related to bacterial growth (Figure 2A). Unlike the complemented Δ*sehB* mutant expressing the SehB-WT protein, none of the point mutants repressed the transcription of the *sehAB-gfp* fusion (Figure 2B). To corroborate the role of SehB mutant proteins on the repression of the *sehAB* bicistronic operon, chromosomal expression of the *sehA* toxin gene was quantified by RT-qPCR. Similar to plasmid reporter fusion, none of SehB mutant proteins were able to complement the levels of *sehA* mRNA compared to the wild-type strain (Figure 2C). Western blotting experiments showed that all His_6_-SehB proteins were produced, indicating that the lack of repressor activity of SehB mutagenic variants was not due to the amount of the proteins (Figure 2D). Our observations show that Y32, L42, L52, I60, S107, L121, L129, and F140 amino acids are relevant for the transcription repression activity of the SehB antitoxin on its own promoter region.

### Amino Acids in Both N- and C-Terminal Are Essential for SehB DNA-Binding Activity

**FIGURE 3:**
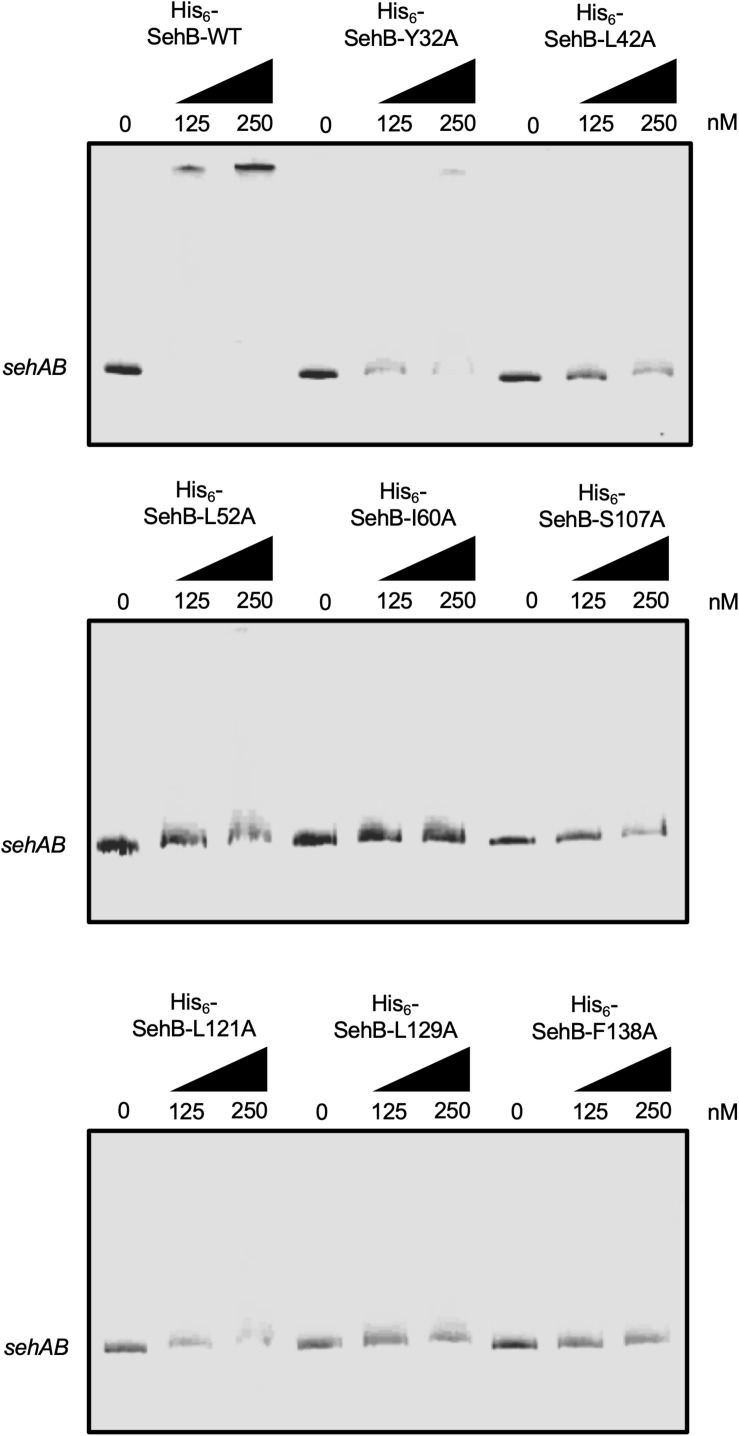
Binding of SehB-WT protein and SehB mutated proteins to the promoter region of *sehAB*. Electrophoretic mobility shift assays were carried out by incubating 100 ng PCR products covering *sehAB* (100 ng) promoter region and increasing amounts (0, 125 and 250 nM) of His_6_-SehB-WT protein and His_6_-SehB mutated as indicated. The complexes were separated on 6% polyacrylamide gels and DNA was stained with ethidium bromide.

SehB represses its own expression by direct binding to its promoter region (De la Cruz et al., 2013). We purified the eight point mutation proteins and wild-type SehB to evaluate their DNA-binding activities. EMSAs were performed using increasing concentrations of the His_6_-SehB proteins and a DNA fragment encompassing the promoter region of *sehAB* (−361 to +20 with respect to the putative transcriptional start site). His_6_-SehB-WT antitoxin was able to bind to its own promoter region at 125 nM and evident DNA-protein complex was detected at 125 and 250 nM (Figure 3) as previously described (De la Cruz et al., 2013). Based on the HTH DNA-binding domain found at the C-terminal, we predicted that mutations in this region may be affected regarding their ability to interact with DNA. Indeed, the DNA-protein complex was not observed with any of the SehB mutants indicating that mutations S107, L121, L129, and F140 affect the binding activity of SehB to the *sehAB* promoter region (Figure 3). Interestingly, the four mutations at the SehB N-terminal were also affected regarding DNA-binding activity (Figure 3), although SehB-Y32A protein still bound to the *sehAB* promoter at 250 nM, leading to the formation of a weak DNA-protein complex. These *in vitro* results show that changes in amino acids located in both N- and C-terminal alter the ability of SehB antitoxin to bind to its own promoter region, which could affect its repressor activity.

### Residues at the N-Terminal of the SehB Antitoxin Are Required for Homodimerization

**FIGURE 4:**
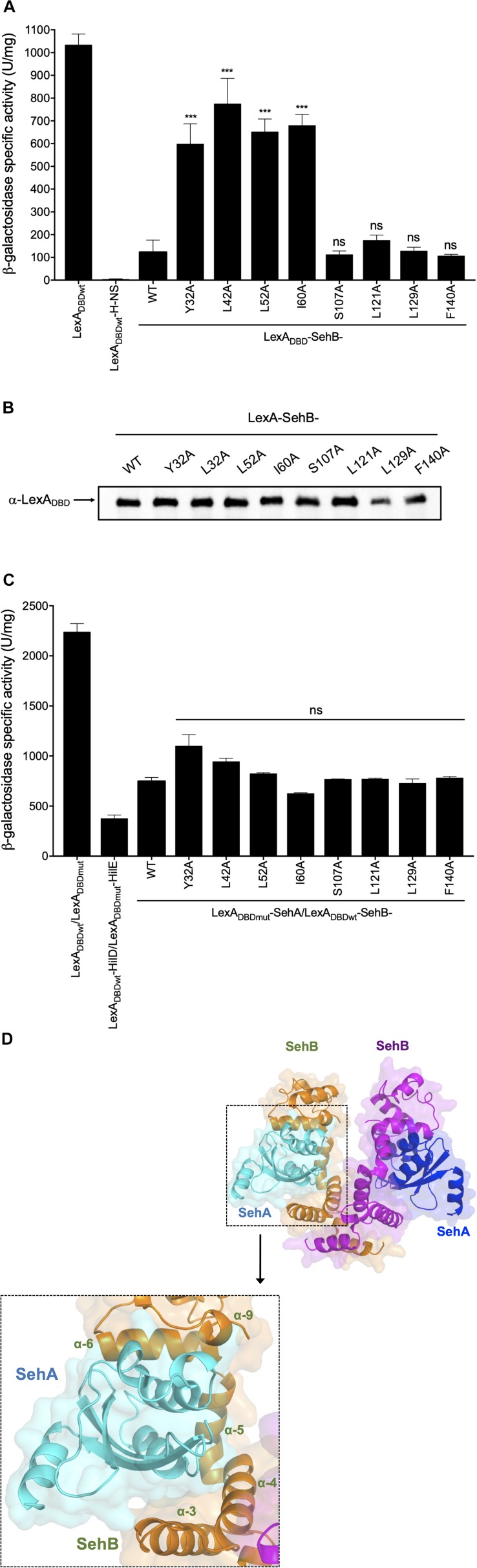
Relevance of SehB amino acids residues in the formation of SehB homodimers and SehB-SehA heterodimers. **(A)** Expression of the *sulA–lacZ* fusion was determined in the *E. coli* SU101 reporter strain containing the plasmids pSR658 (LexA_DBDwt_), pSR658-H-NS (LexA_DBDwt_-H-NS), pSR658-SehB-WT (LexA_DBDwt_-SehB-WT), and pSR658-SehB-point mutations (LexA_DBDwt_-SehB-point mutations). **(B)** LexA_DBDwt_-SehB-WT and LexA_DBDwt_-SehB-point mutations fusion proteins were analyzed by Western Blotting using a polyclonal anti-LexA antibody. **(C)** Expression of the *sulA–lacZ* fusion was determined in the *E. coli* SU202 reporter strain containing the plasmids pSR658 (LexA_DBDwt_), pSR659 (LexA_DBDwt_) pSR658-HilD (LexA_DBDwt_-HilD), pSR659-HilE (LexA_*DBDmt*_-HilE), pSR658-SehB (LexA_DBDwt_-SehB), pSR659-SehA (LexA_*DBDmt*_-SehA), and pSR658-SehB-point mutations (LexA_DBDwt_-SehB-point mutations). The β-Galactosidase activity was determined from samples collected of bacterial cultures grown in LB broth at 37°C to an OD_600 nm_ of 1.0. Expression of LexA_DBDwt_ and the LexA_*DBDmt*_ fusion proteins was induced by adding 1 mM IPTG to the medium. **(D)** Model of SehA-(SehB)_2_-SehA heterotetrameric complex is shown by superposition of HigBA complex structure using PyMol software. SehB antitoxins are colored in orange and magenta, and SehA toxins are in cyan and blue. Zoomed view of toxin-antitoxin interaction domain is showed. SehB α3-α4-α5-α6 are depicted in the 3D-model. Data are the averages of three independent experiments performed by triplicate. The bars represent the standard deviations. ***p < 0.001; ns, not significant.

We have previously reported that the SehB antitoxin forms a stable homodimer in solution or bound to DNA (De la Cruz et al., 2013). In order to corroborate this description, the LexA_DBD__wt_-based genetic system (Dmitrova et al., 1998; Daines and Silver, 2000) was used to analyze SehB homodimerization. LexA_DBD__wt_-SehB-WT repressed the expression of *sulA-lacZ*, confirming that the SehB antitoxin forms an active homodimer (Figure 4A). We used the LexA_DBD__wt_-alone and LexA_DBD__wt_-H-NS fusion proteins as negative and positive controls, respectively (Paredes-Amaya et al., 2018). While changes at the C-terminal were not severely affected in homodimer formation, mutations in the N-terminal domain were defective in their ability to repress the expression of *sulA–lacZ* compared to LexA_DBD__wt_-SehB-WT, suggesting that these amino acids of the N-terminal part are involved in SehB antitoxin dimerization (Figure 4A). All LexA_DBD__wt_-SehB fusion proteins were detected by Western Blotting assays, supporting the notion that differences found in homodimerization of LexA_DBDwt_-SehB mutant proteins was not due to the lack of protein production (Figure 4B). Our data indicate that amino acid residues Y32, L42, L52, I60 of SehB are involved in homodimerization of the antitoxin and subsequently in its DNA binding activity.

### SehB Amino Acid Changes Are Dispensable for the Interaction With the SehA Toxin

In addition to repress its own expression, SehB neutralizes the SehA toxin activity by protein-protein interaction (De la Cruz et al., 2013). To investigate if SehB point mutations were affected in the interaction with SehA, we used the LexA-based genetic system for heterodimerization (Dmitrova et al., 1998; Daines and Silver, 2000). LexA_DBDwt_/LexA_DBDmut_ and LexA_DBDwt_-HilD/LexA_DBDmut_-HilE fusion protein pairs were used as negative and positive controls, respectively (Paredes-Amaya et al., 2018). LexA_DBDwt_-SehB-WT and LexA_DBDmut_-SehA fusion proteins repressed the expression of *sulA-lacZ*, corroborating the direct interaction between SehA and SehB, which is a hallmark of type II TA systems (Figure 4C). None of the SehB point mutants were affected in the interaction with SehA toxin, as they still repressed the transcription of the *sulA-lacZ* fusion (Figure 4C). In fact, the 3D-structure prediction of SehA-(SehB)_2_-SehA heterotetrameric complex revealed that α3, α4, α5, and α6 of each SehB monomer would be interacting with one SehA toxin, suggesting that the SehB-SehA interaction is more complex and it involves more than just one amino acid (Figure 4D). These observations further indicate that single mutations in Y32, L42, L52, I60, S107, L121, L129, and F140 amino acids were not enough to alter the ability of SehB antitoxin to bind SehA toxin.

### SehB Amino Acid Substitutions Affect *Salmonella* Intracellular Replication

**FIGURE 5:**
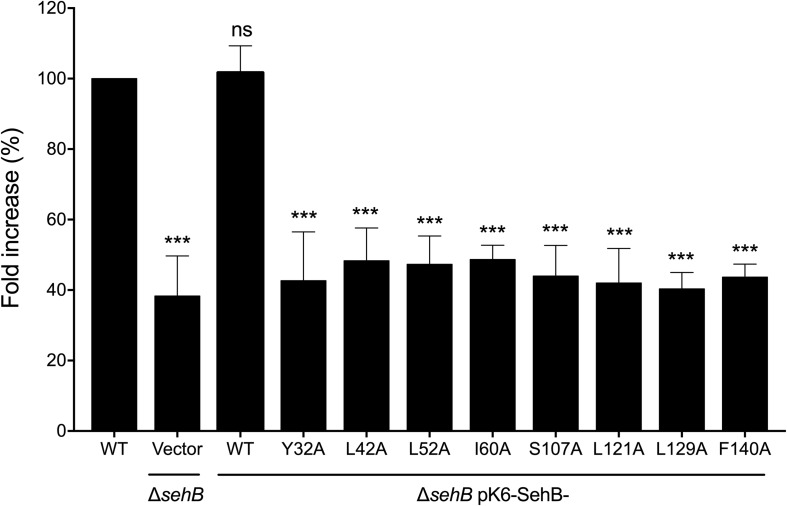
SehB amino acids requirement for the intra-macrophagic replication. RAW264.7 macrophages were infected with *S*. Typhimurium wild-type (WT), Δ*sehB* pMPM-K6 and Δ*sehB* expressing the SehB-WT and SehB point mutations and lysed at 2 h and 16 h p.i. for quantification of intracellular bacteria. The values shown represent the fold increase calculated as a ratio of the intracellular bacteria between 16 and 2 h and normalized to that of the wild-type strain. Values are means ± SD (*n* = 6). The data are averages of three independent experiments performed by triplicate. The bars represent the standard deviations. ***p < 0.001; ns, not significant.

As the absence of the SehB antitoxin affects intracellular replication of *S*. Typhimurium within macrophages (De la Cruz et al., 2013), the role of SehB point mutations in intracellular *Salmonella* replication was tested in RAW264.7 mouse macrophages. Macrophages were infected with either WT *S*. Typhimurium or mutant bacteria expressing the SehB mutant variants, and the fold increase of intracellular *Salmonella* was calculated between 2 and 16 h, compared to wild-type strain. All SehB mutants showed a reduced level of intra-macrophage replication compared wild-type and complemented Δ*sehB* strains (Figure 5). We concluded that Y32, L42, L52, I60, S107, L121, L129, and F140 amino acid residues of SehB antitoxin are required for the *S*. Typhimurium intracellular growth.

### Amino Acids of the SehB Antitoxin Are Involved in *Salmonella* Virulence in Mice

**FIGURE 6:**
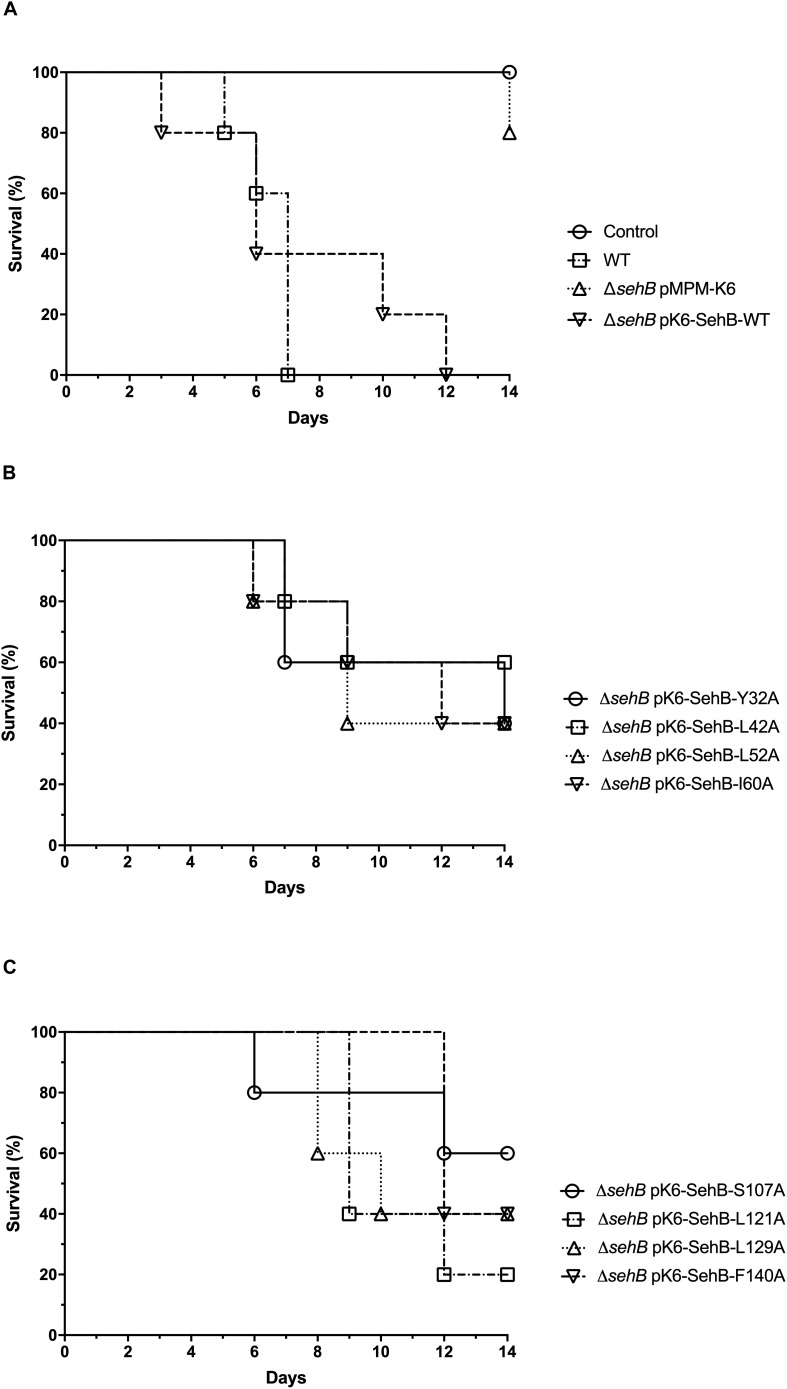
Attenuation of *S*. Typhimurium Δ*sehB* expressing SehB point mutations. Six Balb/c mice were intragastrically infected with 10^5^ CFUs of *S*. Typhimurium wild-type (WT), Δ*sehB* pMPM-K6 and complemented Δ*sehB* mutant (expressing SehB-WT) **(A)** and SehB point mutations at N-and C-terminal **(B,C)** and they were observed during 14 days. The percentages of surviving mice are shown for each strain.

**FIGURE 7:**
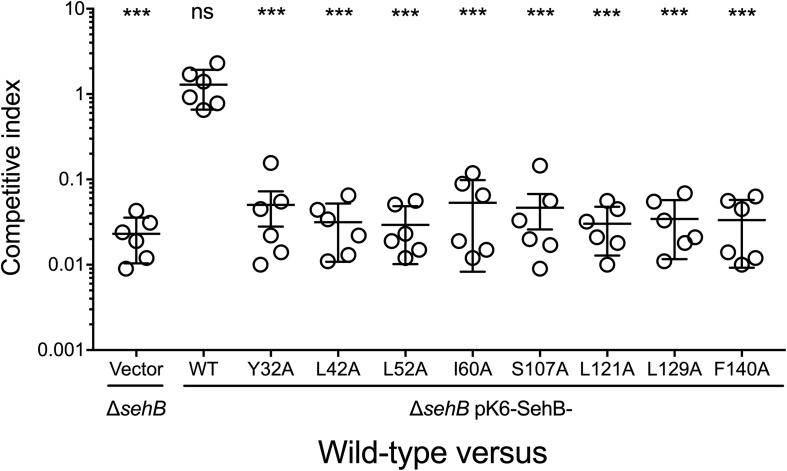
Role of SehB point mutations on the colonization in mice. Six Balb/c mice were inoculated intraperitoneally with a 1:1 mixture of two *Salmonella* strains as indicated. Spleens were harvested 2 days post-inoculation and bacteria were enumerated. Each symbol represents a mouse and horizontal bars correspond to the means ± SD. An unpaired *t*-test was used to determine whether two values were significantly different. ***p < 0.001; ns, not significant.

We previously showed that the absence of SehB antitoxin dramatically affected the *S*. Typhimurium virulence in the mouse model (De la Cruz et al., 2013). To evaluate the contribution of the eight amino acid residues of SehB on virulence, the Δ*sehB* mutant expressing wild-type or mutagenic variants of SehB were tested in survival experiments in which groups of Balb/c mice were infected by gavage with 10^5^ bacteria. These strains were compared to the wild-type (virulent) and to the Δ*sehB* pMPM-K6 strains (non-virulent) strains (Figure 6A). Δ*sehB* strains expressing SehB point mutations appeared partially attenuated in the mouse model. While WT *S*. Typhimurium killed all mice at day 7 p.i., the SehB point mutations located at N-terminal only killed 50% of mice at 14 days (Figure 6B). Changes of amino acids at C-terminal of SehB also attenuated the *S*. Typhimurium virulence (Figure 6C). In addition, the SehB mutagenic variants were tested for virulence by competitive index (CI) experiments. In this assay, each SehB point mutant was co-administered with WT strain to Balb/c mice and the number of each bacterium from the spleen was determined 2 days after inoculation by gavage. In contrast to the Δ*sehB* strain complemented with wild-type SehB, both N-, and C-terminal SehB mutations were poorly recovered from the spleen (Figure 7), showing a CI between 0.1 and 0.01. In conclusion, analysis of the different SehB mutants showed that all SehB amino acids tested are required for *Salmonella* virulence in mice.

## Discussion

During evolution, *Salmonella enterica* acquired and integrated various genetic elements into its genome, which conferred fast adaptive advantages in specific ecological niches. In this context, the type II TA systems form part of the bacterial mobilome and they can be found in both plasmids and chromosomes. *S*. Typhimurium chromosome encodes one of the type II TA systems named SehAB, which is homologous to HigBA family, and is required for the virulence of this bacterium (De la Cruz et al., 2013). HigBA-type TA systems are highly prevalent in bacteria, followed only by the VapBC and RelBE-like TA systems (Pandey and Gerdes, 2005). Of these, HigA-type antitoxins differ mainly from two other type II antitoxins: (i) genetically, the antitoxin gene is located after the toxin gene, and (ii) the DNA-binding domains are mostly located at the C-terminal of the protein (Chan et al., 2016). Interestingly, in contrast to most of the type II antitoxins, the absence of the HigA-type antitoxins seems to be lethal in different bacteria or at least to affect their growth (Budde et al., 2007; Fivian-Hughes and Davis, 2010; De la Cruz et al., 2013; Wood and Wood, 2016). In terms of the regulatory mechanisms, antitoxin components seem to be more complex than those of toxin because the first one controls expression of the second at two levels, transcriptional and post-translational, by directly repressing its own expression and neutralizing *via* protein-protein interaction, respectively (Lobato-Marquez et al., 2016). This functional versatility highlights the importance of characterization of this protein, mainly as a target for molecules that affects its antagonistic activity against the toxin, which results in inhibition of bacterial growth.

Our *in silico* analysis showed that the SehB antitoxin contained two domains, N- and C-terminals, having four α-helices each and these are connected by one α-helix, which functions as a linker. In solution, SehB was detected forming a dimer, bound or not to DNA containing two palindromic sequences found on the −35 and −10 putative boxes (De la Cruz et al., 2013). A dimer of SehB is required for the transcriptional auto-repression, such as was reported for HigA antitoxin in *E. coli* (HigAEco), *S. flexneri*, (HigASfl), *V. cholerae* (HigAVch), *P. aeruginosa* (HigAPae), and *P. vulgaris* (HigAPvu) (Yang et al., 2016; Hadzi et al., 2017; Schureck et al., 2019; Xu et al., 2019; Yoon et al., 2019). Interestingly, the four HigA reported structures show some differences, mainly in structure, organization and location of both dimerization and DNA-binding domains. HigAPae and HigAPvu present a DNA-binding domain similar to HigAEco, although the DNA-binding and dimerization domains are located at the N- and C-terminal, respectively (Schureck et al., 2014). HigAVch protein is more related to *E. coli* RelB antitoxin, possessing both dimerization and DNA-binding domains at the C-terminal (Hadzi et al., 2017). HigAEco and HigASfl antitoxins were reported to have dimerization and DNA-binding domains located at the N- and C-terminals, respectively (Yang et al., 2016; Xu et al., 2019; Yoon et al., 2019). Similar to both HigAEco and HigASfl, which show 46% identity and 67% similarity with SehB, intact N-and C-terminal domains of SehB were required for self-interaction and for DNA-binding, respectively. Although the crystallographic structure of the SehB antitoxin has not been yet resolved, a 3D-model generated using the HigA structures from *E. coli* and *S. flexneri* allowed to observe an homodimer formation (Yang et al., 2016; Xu et al., 2019; Yoon et al., 2019). We propose that L42 and L52 amino acids are participating in the intermolecular contact between both SehB monomers. In contrast, the proximity of Y32 and I60 residues, located at helices α-3 and α-4, respectively, could confer a conformational stability to each SehB monomer, which is required for the homodimer formation. The C-terminal, S107, L121, and L129 amino acid residues form part of the HTH motif necessary for the interaction between SehB antitoxin and its own promoter region. The F140 residue that is conserved in homologous proteins, seems to be interacting with helix α-6 and it probably results in a structure organization required for the conformation of the C-terminal domain, which contains the DNA-binding domain of the antitoxin. In this way, to avoid dramatic changes on the SehB structure, proline or charged amino acids were not selected to be mutagenized in this initial research.

Interestingly, none of the SehB point mutations tested affected heterodimerization between SehB and SehA, keeping the antitoxin neutralizing activity on the toxin. Our 3D-model showed that both homodimerization and toxin interaction domains of SehB are located at the N-terminal, which is similar to what has been reported for HigAEco and HigASfl antitoxins (Yang et al., 2016; Xu et al., 2019; Yoon et al., 2019). Two-hybrid assays and 3D-structure prediction suggest that SehB-SehA interaction is more complex that SehB-SehB, because single mutations on both N- and C-terminal of SehB antitoxin mainly affected the SehB interaction with itself and not the heterodimerization with SehA toxin. Those mutagenic variants still kept the SehA neutralizing activity at the post-translational level despite the lack of repressor activity, explaining why all SehB point mutations were able to complement the growth defect of the *sehB* chromosomal mutant in LB broth. In *E. coli* and *S. flexneri*, HigBA toxin-antitoxin complex has higher affinity to DNA than HigA antitoxin alone (Yang et al., 2016; Yoon et al., 2019). However, HigA alone presents higher affinity to DNA than HigBA complex in *P. vulgaris* (Schureck et al., 2019). We were unable to analyze if SehAB presented higher or lesser DNA-binding affinity than SehB alone because the SehA-(SehB)_2_-SehA complex was not possible to reconstitute using both purified proteins, as previously described (De la Cruz et al., 2013).

In the absence of SehB antitoxin, high levels of SehA toxin are expressed, partially affecting the bacterial growth in LB broth resulting in severe attenuation of virulence in the mouse model (De la Cruz et al., 2013). We proposed that in addition to SehA toxin, other elements present in the *S*. Typhimurium genome, could be affected in the absence of SehB antitoxin, mainly to transcriptional level. This hypothesis is supported by reports where the absence of HigA antitoxin in *P. aeruginosa* transcriptionally affects other genes that encode for virulence factors, such as pyochelin, pyocyanin, type III secretion system, MvfR transcriptional regulator, and those related with the c-di-GMP metabolism (Li et al., 2016; Wood and Wood, 2016; Zhang et al., 2018; Guo et al., 2019).

The contribution of SehB to *S*. Typhimurium virulence allowed the identification of amino acid residues involved in the intracellular replication and colonization in mice. According to what is observed in the ability of homodimerization and DNA-binding, all amino acids tested in this study were relevant for the functioning of SehB and needed to confer virulence to *S*. Typhimurium. Survival curves and competitive indices experiments in mice showed that Y32, L42, L52, I60, S107, L121, L129, and F140 amino acid residues are required for *S*. Typhimurium virulence. Although Δ*sehB* mutant expressing pK6-SehB-WT fully complemented the virulence of *Salmonella* in macrophages and competitive indices assays, this complementation was partial in the mice survival curves. Since pK6-SehB-WT plasmid was stable during the 14 days of mice monitoring (data not shown), we hypothesized that when the bacterium senses some signals at late times during the systemic infection (i.e., after 6 days), the expression of arabinose promoter from plasmid (which drives the *sehB* transcription) could be down-regulated, or may mechanisms related to copy number or segregation be affected.

The importance of each amino acid residue could be explained by the small size of the SehB antitoxin and the complexity of its regulatory function. Although SehB antitoxin neutralizes the SehA toxin activity by protein-protein interaction, the primary control on the *sehAB* expression is based in the transcription activity of SehB on its own promoter region. Future work will be aimed to design molecules that function as drugs in order to affect either homodimerization capacity or SehB DNA-binding.

## Data Availability Statement

The raw data supporting the conclusions of this article will be made available by the authors, without undue reservation, to any qualified researcher.

## Ethics Statement

The animal study was reviewed and approved by the Internal Ethics Committee of the Animal Resource Facility of the Universidad Autónoma del Estado de Hidalgo (Approval number: CIECUAL/002/2018 to Manuel Sánchez-Gutiérrez).

## Author Contributions

MD conceived and designed the experiments. FC-C, GH-M, SP, MA, and JS-B performed the experiments. MD, MA, MS-G, JI-V, JI, JG, J-PG, SM, and MD analyzed the data. MD wrote the manuscript.

## Conflict of Interest

The authors declare that the research was conducted in the absence of any commercial or financial relationships that could be construed as a potential conflict of interest.
